# Mediterranean Diet and Health Outcomes in the SUN Cohort

**DOI:** 10.3390/nu10040439

**Published:** 2018-03-31

**Authors:** Silvia Carlos, Carmen De La Fuente-Arrillaga, Maira Bes-Rastrollo, Cristina Razquin, Anaïs Rico-Campà, Miguel Angel Martínez-González, Miguel Ruiz-Canela

**Affiliations:** 1 Department of Preventive Medicine and Public Health. University of Navarra. Pamplona 31008, Spain; mbes@unav.es (M.B.-R.); crazquin@unav.es (C.R.); aricoc@unav.es (A.R.-C.); mamartinez@unav.es (M.A.M.-G.); mcanela@unav.es (M.R.-C.); 2 IdiSNA, Navarra Institute for Health Research, Pamplona 31008, Navarra, Spain; 3 Centro de Investigación Biomédica en Red Área de Fisiopatología de la Obesidad y la Nutrición (CIBEROBN), Madrid28029, Spain

**Keywords:** Mediterranean Diet, adherence, chronic disease, cardiovascular, diabetes, weight, metabolic, depression, cognitive decline, nephrolithiasis

## Abstract

The Mediterranean Dietary (MedDiet) Pattern has been linked to many beneficial health effects. This review summarizes the main findings of a prospective cohort study, the Seguimiento Universidad de Navarra (SUN) cohort, specifically focused on MedDiet and the risk of major chronic disease. It is an open cohort in which 22,786 Spanish university graduates have participated since 1999 until February 2018. Data on diet, lifestyle and clinical diagnosis are collected at baseline and every two years. After reviewing 21 publications from the SUN cohort on the effects of the MedDiet, we conclude that this cohort has provided good evidence that a high MedDiet adherence is associated with a reduced incidence of all-cause mortality, fatal and non-fatal major cardiovascular disease (CVD), type 2 diabetes, weight gain, metabolic syndrome, depression, cognitive decline, and nephrolithiasis. An inverse dose-response relationship was found for many of these associations. The MedDiet was also associated with lower average heart rate, a mitigation of the harmful effects of overweight/obesity on the risk of CVD, and an attenuation of the effects of obesity on type 2 diabetes. A suggestion that the MedDiet may enhance fertility was also found.

## 1. Introduction

Today nutritional epidemiology is mainly focused on food patterns rather than on individual foods or nutrients. Dietary patterns combine different food intakes and take also into account the interactions between different food groups and their synergistic or antagonistic effects. 

Among different dietary patterns assessed in longitudinal studies, the Mediterranean Diet (MedDiet) has been linked to many beneficial health effects (decreased risk of all-cause mortality, cardiovascular disease, cancer, and other chronic diseases) [1]. This pattern is defined using olive oil as the main source of fat, a high consumption of plant-derived foods (i.e., fruits, nuts, vegetables, legumes, cereals, and seeds), frequent consumption of fish, moderate wine consumption with meals, and low consumption of meat (mainly poultry), dairy products and sweets. Since the MedDiet was first described, different scores have been proposed for measuring this dietary pattern [2,3]. One of the most frequently used scores is the Mediterranean Diet Score (MDS) defined by Trichopoulou, which is a 0- to 9-point scale that uses sample sex-specific medians of food groups to define these points [4]. Apart from the dietary aspects, other sociocultural and structural aspects related to this food pattern could contribute to the MedDiet beneficial effects [5].

Prospective cohorts are needed to evaluate the effects of MedDiet on the incidence of major diseases such as those chronic diseases which are today the main public health problems worldwide. In this sense, the SUN cohort is a large prospective cohort of relatively young adults at baseline which is ongoing since 1999 and which collects and updates detailed information on diet, dietary changes, and lifestyle as well as on the incidence of a wide variety of chronic diseases. A major strength of the SUN cohort is that it was specifically designed to assess the effects of the MedDiet in a Mediterranean setting. In this manuscript, we summarize the main findings of the SUN cohort study with regards to the MedDiet (mainly evaluated with the MDS proposed by Trichopoulou) and the incidence of major chronic disease.

## 2. The SUN (Seguimiento Universidad De Navarra) Study: Design

The SUN cohort is a multipurpose cohort focused on the evaluation of the impact of diet and lifestyle on the prevention of non-communicable diseases (diabetes, gestational diabetes, cancer, cardiovascular disease, ophthalmological, reproductive, mental, respiratory diseases, and injuries). Among other factors, the main objective of the SUN cohort is to study the benefits of the Mediterranean Food Pattern.

### 2.1. Study Participants

The SUN study is a dynamic, permanently open prospective cohort. Since December 1999 graduates from the University of Navarra as well as from other different Spanish universities, all aged 20 years and over, are invited to participate [6,7]. This selection of highly educated participants corresponds to the approach known as restriction in epidemiology and it was applied to control for confounding by socioeconomic status. As explained by Rothman et al., restriction is “an excellent technique for preventing or at least reducing confounding by known factors” [8].

### 2.2. Data Collection

#### Questionnaires

At baseline, once the participants accept to enter the study, they receive a detailed questionnaire by ordinary mail or an email with a personal code to answer the questionnaire at the SUN website (this second alternative is available since 2004). Less than 2% of participants has used the online system to answer the baseline questionnaire.

This baseline questionnaire collects information on participants’ sociodemographics, anthropometrics, diet, eating behaviors, lifestyles, clinical data, preventive measures, medications, and personality traits (Table 1). 

In the baseline questionnaire, diet is assessed using a 136-item semi-quantitative food frequency questionnaire (FFQ). Each item of the FFQ shows a typical portion size. Daily intake is calculated by multiplying the portion size by the frequency of consumption (nine options ranging from never/almost never to six times or more/day). Nutrient intake is estimated using Spanish food composition tables [9,10] and other sources of information [11]. As in other well-known nutrition cohorts, such as in the Nurses’ Health Study [12] or the Cancer Prevention Study II Nutrition Cohort [13], the SUN cohort FFQ has been repeatedly validated in Spanish participants [14,15,16] and it has been also shown to correlate well with biomarkers of intake [17]. Other self-reported items of the questionnaires have also been validated: anthropometric data [18], physical activity [19], hypertension [20], metabolic syndrome [21,22] and depression [23,24]. Anthropometric measurements are self-reported and when measurements are requested (after 6 and 8 years), a measuring tape and instructions are mailed with the questionnaire to allow the participants to measure their waist and hip circumferences.

### 2.3. Follow-up

Every two years, shorter follow-up questionnaires are sent by ordinary mail or e-mailed to track changes in food habits, diagnosis of new diseases, and overall well-being (Table 1). In many (but not all) follow-up questionnaires there are a few questions on diet. In the 10-year follow-up questionnaire the full-length validated FFQ is repeated. The percentage of questionnaires filled-in online increases in the follow-up questionnaires in comparison with the baseline questionnaire and from Q10 it is higher the number of participants who uses the online than the questionnaire in paper. 

The follow-up is calculated from date of returning the baseline questionnaire to date of death, date of the incident disease under study or date of returning the last follow-up questionnaire for participants who did not develop the disease. For each participant, three postal addresses, an email and phone numbers are requested. Up to five additional mailings are sent to non-respondents, after which a brief questionnaire is mailed and finally, they are contacted by email or phone. Finally, in case participants do not answer, alumni associations and other professional associations are inquired to obtain the contact of participants. For lost to follow-up participants, to confirm deaths and their causes, we check every year both the Spanish National Death Index (S-NDI) and the National Statistics Institute (NSI) (please see [25] for a comparison between the two official Spanish sources of information on mortality). The S-NDI was started relatively recently in Spain (2000) and it is the standard source of data for assessing deaths in cohort studies in Spain. The access to the NSI, which is considered more accurate, allows the use of microdata with personal identifiers capable of linking the NSI databases with research files. This is only possible after a rather arduous process, including the signing of an agreement between the NSI and our institution (the University of Navarra in our case). The research databases need to be submitted in a specified format, there is a need to pay the stipulated price and, finally, there is a step involving a collaborative work between one member of the research team and officials of the NSI to decide on listings whether a death can be assigned to cases with partial agreement of personal identifiers. We follow all the procedures required.

The positive predictive value for these sources of information regarding fatal events is very high, expected to be around 100%. 

Families and postal authorities also report deaths, which are confirmed by a review of medical records (with permission of the next of kin). 

In February 2018, the overall retention rate in the cohort was 91% (any follow-up), 88% at Q2 (*n* = 18,932), 81% at Q4 (*n* = 17,200), 82% at Q6 (*n* = 15,327), 81% at Q8 (*n* = 13,863), 81% at Q10 (*n* = 12,234), 84% at Q12 (*n* = 10,145) and 78% at Q14 (*n* = 6,024). During this 18-year period, we have lost contact with 263 participants, 1,008 have explicitly requested to cancel their participation in the cohort for different reasons and 384 participants have died. However, as Alonso et al. reported differences in follow-up in the SUN cohort did not seem to cause a bias and did not necessarily affect the magnitude or direction of the association estimates [26,27].

#### Biorepository

The collection of biological samples began in 2008. Saliva samples were requested from all participants aged 55 years and over, for a substudy on dementia. In this substudy, cognitive function was evaluated by the Spanish phone version of the Mini Mental State Examination, the modified Telephone Interview for Cognitive Status (TICS-m) test. Telephone interviews are recorded as follows: initial, 2-year, 4-year, and 6-year. Saliva samples are collected at the initial examination.

A commercial kit for the sample collection (Oragene DNA OG-500 saliva extraction kit from DNA Genotek (Ottawa, Canada) is sent by ordinary mail to the participants that accept to participate. The kit includes an all-in-one system for the collection, stabilization, and transportation of the saliva. Once the samples were received at the Preventive Medicine Department, they were kept at 4 °C until the DNA extraction was carried out. The extracted DNA was then kept at −80 °C. For the analysis of the different genetic variants in candidate genes as APOE4, CLU, CR1, FTO and PICALM among others, real-time PCR followed by an allelic discrimination was carried out [28,29].

Until December 2017, 1,500 samples had been collected from participants that had also answered to the dementia-related interview (TICS-m). 

### 2.4. Data Management and Statistical Analysis

#### 2.4.1. Database

All data are electronically entered and exported to a secure web-based database.

##### 2.4.1.1. Mediterranean Diet Adherence

In the different analyses that have been carried out since the beginning of the SUN study, the most frequently used score in the SUN study for the evaluation of the adherence to the MedDiet is that proposed by Trichopoulou et al. [4]. It assigns a score of 0 or 1 to each of the following 9 diet components: (1) high fruit and nuts intake; (2) high vegetables intake; (3) high cereals intake; (4) high legumes intake; (5) high fish intake; (6) high MUFA:SFA ratio; (7) moderate alcohol intake (5–25 g/day for women or 10–50 g/day for men); (8) low meat intake; and (9) low dairy intake. One point is assigned to consumptions of the beneficial components (vegetables, fruits/nuts, legumes, fish/seafood, cereals, and monounsaturated/saturated fatty acids ratio (MUFA/SFA)) at or above the sex-specific median. Then, this score (0–9) has been categorized into different groups depending on the study but usually as 0–2, 3–6 and 7–9. 

Only a few studies within the SUN project have used an a posteriori approach for measuring the MedDiet adherence [30,31,32,33]. 

##### 2.4.1.2. Outcomes

Up to now the outcomes that have been evaluated in the SUN study related to MedDiet are: all-cause mortality, non-fatal and fatal cardiovascular events (myocardial infarction, stroke, revascularization, cardiovascular death, hypertension, heart rate), type 2 diabetes, gestational diabetes, weight gain/change, overweight/obesity, metabolic syndrome, depression, cognitive decline (evaluated by means of TICS-m changes), self-perceived quality of life, difficulty in conceiving and nephrolithiasis.

#### 2.4.2. Statistical Tests

All studies include descriptive and multivariate analyses, and mostly carried out using Cox or logistic regressions.

#### 2.4.3. Sensitivity Analyses

Most studies of the SUN project have conducted sensitivity analyses additionally using alternative definitions of the Mediterranean diet and in most of them the reported associations remained. 

## 3. Results

### 3.1. Participation. Response Rates

Since 1999 until February 2018, 22,786 participants have participated in the SUN study and the number of participants is constantly increasing. 

Among all participants who are offered to participate in the study only 10% have accepted to enter. We used hurdles to participation to selectively recruit highly motivated participants, as recommended [34].

### 3.2. Characteristics of Participants 

Overall, most participants are young adults. The median age is 35 years (interquartile range, IR: 27–45) and 82% younger than 50 years. The majority are women (61%). Most of them are married (50%) or single (46%). The majority are graduated in a health-related profession (55%) and have a paid employment (76%).

At entry 25% of all participants are current smokers and 24% are former smokers. The median body mass index is 23 (IR: 21–26) kg/m^2^ and approximately 70% of participants practice regularly some physical exercise. Around 8% of participants report a previous diagnosis of a chronic disease (type 2 diabetes, cardiovascular disease, or cancer) and 13% of depression. 

The main characteristics of study participants according to their baseline adherence to the MDS, adjusted for age and sex, are shown in Table 2. Participants with the highest adherence to the MDS as compared to those in the lowest category are more likely to be health professionals. They are more active physically, more likely to be former smokers and report a significant higher total energy intake, carbohydrate intake and less saturated fatty acids intake. They also report a higher baseline consumption of fiber, alcohol, olive oil, vegetables, fruits, legumes, cereals and fish, and a lower consumption of dairy, meat, soft-drinks, and fast-food. Importantly, the increased consumption of fiber in association with better adherence to the MedDiet, as captured by our FFQ, seems to be an important characteristic explaining a better cardiovascular risk profile [35,36].

With regards to the different outcomes evaluated (Table 3), the incidence of cardiovascular events was lower among participants with a higher adherence to the MDS compared with those with the lowest adherence. Likewise, the incidence of overweight/obesity, metabolic syndrome, depression, and nephrolithiasis was lower in this group. 

### 3.3. Outcomes

All the results found in the SUN cohort related to the effects of the MedDiet on each of the major chronic diseases evaluated are described below and summarized in Table 4.

#### 3.3.1. All-Cause Mortality 

Álvarez-Álvarez et al. analyzed the data from 19,467 participants from the beginning of the cohort until February 2013 and found that a better adherence to the MedDiet was significantly associated with a lower risk of all-cause mortality [1]. For the MDS-Trichopoulou, they did not observe statistically significant differences between extreme tertiles, but a statistically significant inverse linear trend was found. When the increase in 2 points in the MDS was analyzed, the effect was marginally significant. A better adherence to the MDS combined with a more physically active lifestyle showed a strong inverse association with all-cause mortality (the strongest association was observed for vigorous activities together with the best adherence to the MDS) (HR = 0.29, 0.13–0.65) (supplementary data). In this study, the consumption above the median of fruit, or below the median of meat products was inversely associated with all-cause mortality. 

Before this study, Zazpe et al. also found a lower risk of all-cause mortality for participants of the SUN cohort with the highest adherence to the MedDiet (HR: 0.53, 95% CI 0.34–0.84), with a significant linear trend [30]. They used a post hoc score based on factor loadings of the different food groups using principal components analysis. When sensitivity analyses were done, the association between a better adherence to MedDiet and lower mortality was only present among health professionals and only in men (maybe due to the fewer deaths observed in women).

Domínguez et al. analyzed in 2013 the effect of the adherence to MDS-Trichopoulou on a composite outcome that included all-cause mortality, CVD, and type 2 diabetes (because the majority of the participants in the cohort were young adults and there were few events when they analyzed the separate components). They found that adherence to the MDS was associated with a decreased incidence of this composite outcome (OR: 0.81, 0.69–0.95, per 2 points) [37].

Finally, Martínez-González and cols. also analyzed in 2012 the association of the MDS with mortality and found an inverse association between adherence to the MDS and the risk of death and it remained significant in most sensitivity analyses. Each 2-point increment in the adherence score was associated with a 28% relative reduction in all-cause mortality. The inverse association was stronger for men and for deaths occurring at earlier ages. The strongest association was found for cardiovascular deaths and they did not find an association with deaths from cancer (only 48 deaths). When the specific foods were evaluated, they found that only the item ‘‘fruit and nuts’’ was independently associated with a lower risk of death among both men and women [38].

A plausible explanation for the inverse association between better adherence to the MedDiet and all-cause mortality is that the food groups that are positively weighted in the MDS contain antioxidant and anti-inflammatory phytochemicals (plant foods) that prevent chronic diseases. They also contain phenolic compounds (virgin olive oil) associated with anti-atherogenic effects and inhibition of oxidative stress, and antioxidant polyphenoles (red wine) protective for cardiovascular disease. Other possible mechanisms could contribute to lower overall mortality: improved indexes of glucose homeostasis, reductions in blood pressure, abdominal obesity, metabolic syndrome, and higher HDL cholesterol levels. Additionally, as explained by Zazpe et al., individuals with a higher adherence to this dietary pattern had a better nutrient profile than those with the lowest adherence [30]. 

#### 3.3.2. Cardiovascular Disease

Eguaras et al. found in 2015 that a better adherence to the MDS was associated not only with reduced CVD (HR = 0.47, 0.25–0.89; *p* for linear trend = 0.029), but also with a mitigation in the harmful effect of overweight/obesity on CVD: within the low MDS adherence group, the risk of CVD increased across categories of body mass index (BMI), while the negative effect of BMI on CVD was attenuated for those with a higher adherence [39]. It seems biologically plausible that the MedDiet anti-inflammatory effects can counter-balance the detrimental effects of the low-grade inflammation associated with obesity.

Martínez-González and cols. described in 2011 an inverse association between adherence to the MDS and the incidence of both fatal and non-fatal CVD [40]. They found that participants with the highest adherence had a 59% significant lower CVD risk than those with the lowest adherence. For coronary heart disease, the difference between extreme categories of adherence rendered similar estimates with a significant trend. In their analyses, fruits and nuts were the only item predictive of a lower CVD incidence although most estimates were in the expected direction, except for cereals. In fact, when a new score without cereals was used, the inverse association with CVD was enhanced. Furthermore, when the association was evaluated specifically for white bread and coronary disease the HR was 1.92 (1.05–3.51, *p* for linear trend = 0.005), although the estimate lost significance when the analysis was adjusted for total energy intake. The MDS proposed by Trichopoulou does not make a distinction between whole grains (protective for CVD) and refined cereals, which lead to a high glycemic load, associated with a decrease in HDL cholesterol, increased fasting plasma triglycerides, higher fasting insulin, and increased levels of C-reactive protein [40].

#### 3.3.3. Hypertension

Núñez-Córdoba et al. found for all categories of adherence compared to low adherence, that adherence to the MDS was not associated with hypertension but it was inversely associated with changes in mean systolic and diastolic blood pressure after 6 years of follow-up [41]. A direct association between hypertension and intermediate alcohol intake (5–25 g/day among women or 10–50 g/day among men) was found (red wine was not specifically evaluated). 

#### 3.3.4. Heart Rate

García-López et al. [42] found that a higher adherence to the MDS was associated with a significant lower average heart rate (linear association), which according to prior studies, could correspond to a lower risk of sudden death [43]. The high fish consumption characteristic of the MDS could have influenced this lowering of the average heart rate, because of increased omega-3 fatty acids intake, because docosahexanoic acid may be responsible for this inverse association. Also, a moderate alcohol consumption is likely to be related to a lower average heart rate, as well as fruit and vegetables which may enhance the parasympathetic response and thus a decreased heart rate.

#### 3.3.5. Type 2 Diabetes

In 2008 Martínez-González et al. found that a high adherence to the MDS was associated with a substantial reduction in the risk of developing type 2 diabetes, with a significant linear trend [44]. These findings initially reported by the SUN cohort, with a small number of cases, were subsequently confirmed by other cohorts [45] and by the PREDIMED intervention trial [46]. One potential mechanism for this effect of MedDiet on diabetes is the fact that virgin olive oil protects against insulin resistance and the metabolic syndrome. The high MUFA content of olive oil might lead to improved insulin sensitivity and to better lipid profiles. In addition, the MedDiet pattern is related to lower plasma concentrations of inflammatory markers and markers of endothelial dysfunction which are predictive of the occurrence of type 2 diabetes. Finally, a high adherence to a MedDiet has been reported by other authors to be associated with higher levels of adiponectin, and this mechanism may also partly explain the reduced diabetes risk [47]. 

Apart from the direct effect of the MedDiet on diabetes, Eguaras et al. have recently shown that MedDiet can attenuate the adverse effects of obesity on the risk of type 2 diabetes [48]. Those SUN cohort participants with a low adherence to the MDS had a significantly higher risk of obesity-associated type 2 diabetes than those with a high adherence, which is also observed when both the MDS and BMI were analyzed as continuous variables. Moreover, the inverse association remained when the models were additionally adjusted for weight changes during follow-up. Therefore, a mitigation of the pernicious effect of obesity on type 2 diabetes incidence by the MedDiet may exist even without substantial weight loss.

#### 3.3.6. Gestational Diabetes

In a sample of 3,455 pregnant women of the SUN cohort with 173 incident gestational diabetes mellitus (GDM) Donazar-Ezcurra et al. found no association between adherence to the MedDiet (empirically derived pattern) and GDM incidence (adjusted OR: 1.08; 95% CI: 0.68–1.70 for the highest quartile versus the lowest) [31]. These results disagree with previously published literature, although they cannot be completely compared as they considered different scores to assess the MedDiet and the populations were different. In addition, the authors explained that this null association might be explained because it was not known whether women with an unhealthy dietary pattern might have changed their diet as a result of planning a new pregnancy. 

#### 3.3.7. Overweight/Obesity, Weight Gain and Metabolic Syndrome

Beunza et al. found in 2010 that the group of participants in the SUN cohort with the highest adherence to the MDS compared to the group with the lowest adherence had the lowest weight gain during the first 4 years of follow-up [49]. They also had the lowest risk of an absolute weight gain >3 or >5 kg during the first 2-year and 4-year follow-up. However, no association was found with the incidence of overweight or obesity. Finally, when an additional analysis was done to evaluate the weight change as percentage of change from baseline, a significant inverse association with adherence to the MDS was found with an inverse linear trend. The high fiber content in the MedDiet, olive oil and nuts may lead to increased satiety. Also, the low energy density and low glycemic load can provide a better metabolic control and a lower calorie intake.

In a previous study Sánchez-Villegas et al. [50] found that participants with a higher adherence to the MDS had smaller weight increments although the statistical significance was lost after multivariate adjustment. 

A higher baseline meat consumption was associated with higher weight gain (linear trend) but a higher consumption of whole-fat dairy products was associated with lower weight gain (linear trend). This later association could be due to intracellular calcium or microbiota-related mechanisms. For a high consumption of the rest of the groups (fruit, nuts, and olive oil) a beneficial effect was apparent but not statistically significant, although nuts, for example, can have a protective effect against weight gain [51] due to the increased resting energy expenditure and their effect on satiety as a consequence of their high content in fiber and proteins [52]. The inverse association between nut consumption and weight gain was subsequently replicated in an independent cohort [53].

Tortosa et al. found that participants with the highest adherence to the MDS had lower incidence of metabolic syndrome compared with the lowest adherence [54].

#### 3.3.8. Depression

Sánchez-Villegas et al. in 2015 found that a better adherence to the MDS was associated with a reduced risk of depression [55]. The magnitude of the association was similar for other high-quality dietary patterns evaluated in the study (Pro-vegetarian Dietary Pattern and the Alternative Healthy Eating Index-2010) [55]. A moderate adherence to the MDS at baseline was associated with an important reduction in the risk of developing depression during the follow-up as compared to the minimum adherence. When changes in the adherence were considered, the association was attenuated although the dose-response relationship remained significant. It seemed to be a threshold effect so that although the risk of depression was reduced when comparing moderate versus lower adherence, there was not much extra benefit for the comparison between moderate and high or very high adherence (*L*-shaped association). The effect could be associated with the intake of some vitamins (e.g., B-vitamins and folate, vitamin E) and minerals (e.g., magnesium or zinc) as well as omega-3 fatty acids or the omega-3/omega-6 ratio could be influencing the observed effect but more evidence on the causal mechanisms is needed.

Sánchez-Villegas et al. had previously analyzed the effect on depression of the MDS and found an inverse association for the upper categories of adherence, with reductions in the risk of depression higher than 30% [56]. The results did not change when the participants’ psychological characteristics were additionally included in the multivariate models. When the individual components of the MDS were assessed a statistically significant linear trend across categories of fruit and nuts, legumes and the MUFA/SFA ratio was observed. Regarding fish consumption, a 20% reduction was observed for intermediate quintiles, without a significant linear trend. On the contrary, for whole-fat dairy and meat a significant linear trend was observed for a direct association with depression. 

As stated by Sánchez-Villegas et al. [56] a better adherence to the MedDiet has been linked with improvements in endothelial cells which synthesize and secrete brain derived neurotrophic factor (BDNF), which is critical for axonal growth, neuronal survival, and synaptic plasticity. Among patients with depression BDNF levels are lower and a potential mechanism of a good MedDiet adherence in lowering depression risk is through the improvement of BDNF production. A high consumption of red wine and olive oil could also improve the endothelial function in healthy individuals. Another possible mechanism is through the reductions in the levels of cytokines and inflammatory modulators as depression is associated with increased proinflammatory cytokines (e.g., IL-1, IL-6 and C-reactive protein), which may inhibit BDNF expression, interfere with neurotransmitter metabolism, and alter neurotransmitter messenger RNA. Olive oil can contribute due to its antioxidant properties and the increase in the delta-9 desaturase enzyme activity maintaining the neuronal membranes physiochemical properties. Finally, B vitamins (B6 and B12) and folate are involved in the synthesis of methionine which is involved in the production of serotonin and other monoamine neurotransmitters which have antidepressant properties. 

Subsequently, Sánchez-Villegas et al. tried to replicate the findings of the SUN cohort in a randomized primary prevention trial (PREDIMED), but the inverse association with depression was not statistically significant [57]. More recently, Sánchez-Villegas et al. have analyzed not only the effect of a higher adherence to MedDiet but the effect of a Mediterranean lifestyle, including a high level of physical activity and social activity [5]. The three exposure variables, both individually and combined, were inversely associated with depression risk with significant dose-response relationships. The level of activity (both physical activity and socializing) could be at least as important as diet to reduce the risk of depression. 

#### 3.3.9. Cognitive Decline 

Galbete et al. found in 2015 that individuals with low and moderate adherence to the MDS showed lower scores in cognitive function than subjects with a higher adherence (no linear trend was observed, suggesting that a threshold effect may exist) [29]. When they analyzed the association excluding the MUFA:SFA ratio from the score and adjusting for this variable, the inverse association disappeared, suggesting that the fatty acid profile of olive oil is important for this association. A low MUFA:SFA ratio was significantly associated with a higher decline of the cognitive function.

The cardiovascular protective effects of the MedDiet may influence cognitive function. With regards to the effect of olive oil, the extra-virgin variety has antioxidant properties that can reduce neuronal damage. As an example, oleocanthal, enhances amyloid-β clearance from the brain [58].

#### 3.3.10. Quality of Life (Self-Perceived Mental and Physical Health)

A study carried out in 2013 by Ruano et al. found that adherence to a MedDiet (calculated with a post hoc approach by factor analysis) was directly associated with better scores in quality of life four years later. Even though most participants perceived themselves in good health, there was a significant direct association with the mental summary component (linear trend) but the association with the physical component was not significant [32]. With regards to the mental health components, omega-3 fatty acids are likely to influence the central nervous system, including the structure and fluidity of neural membrane and serotonin transport. Also, B vitamins and folate are involved in methylation reactions, as those related to serotonin and other neurotransmitter synthesis.

Henríquez et al. had previously analyzed this association but considering the MDS Trichopoulou [59]. They found a significant direct linear association with the physical domain and with three of the mental health domains (vitality, social functioning, and emotional role). An increment in one-point in the score was related to 0.50 (95% CI: 0.32–0.68) point increment in vitality and 0.45 (95% CI: 0.26–0.62) point increment in general health. Furthermore, participants with the highest baseline MDS adherence who increased their adherence during follow-up showed significant increments in the scores of physical functioning, general health, and vitality when they were compared with those with the lowest baseline adherence and who had decreased or maintained their initial adherence.

It is not clear how to define meaningful clinical differences on the SF-36 scores. For almost all the quality aspects, a magnitude of the differences between the lowest and highest adherence of about 2–3 points was found in this study. 

#### 3.3.11. Difficulty Conceiving

Toledo et al. found in a case-control study within the SUN study, that a greater adherence to a Mediterranean-type dietary pattern may enhance fertility [33]. Women with the highest adherence to MedDiet (empirically derived) compared with those with the lowest adherence showed significantly lower odds of consulting a physician because of difficulty getting pregnant. 

#### 3.3.12. Nephrolithiasis

Up to now few studies have examined the impact of dietary patterns on the risk of nephrolithiasis and particularly, there were no studies regarding the MedDiet. Leone et al. have recently found an inverse significant association for a high adherence to the MDS with an almost 40% relative risk reduction and a significant inverse dose-response trend [60]. In addition, when they additionally took into account changes in the adherence to the MDS, the association remained significant. For vegetables and dairy there was a significant inverse dose-response relationship. There was an inverse association for the higher quintiles of fruit and nuts, but the linear trend was not significant. Unexpectedly, they found a positive association between MUFA: SFA ratio and the risk of nephrolithiasis.

Although a higher consumption of vegetables, fruits, and nuts may increase urinary oxalate associated with calcium oxalate nephrolithiasis, a higher fruit and vegetable intake can be also associated with a higher fluid intake and therefore urine dilution, as well as increase some stone inhibitors, such as pH, potassium, magnesium, fiber, citrate or phytates which can reduce the supersaturation of calcium. With regards to dairy, against the classical belief, calcium could reduce the risk of nephrolithiasis as more calcium in the intestine can reduce the absorption and thus, the excretion of oxalate [60]. There are yet no data on the association between MUFA intake and the risk of nephrolithiasis.

### 3.4. Mechanisms Through Which the Mediterranean Diet Works 

Today a lot of work is being done to understand the individual or shared mechanisms that can explain the inverse association between MedDiet and each of the different chronic diseases. It is a complex evaluation considering that the ethiopathology takes place at varied different levels (molecular, cellular, tissue, and organ), that there are many pathways common to different diseases and that there are synergetic effects between the different food components, the ways of cooking, the environment, the physical activity, and many other known and unknown factors. Different mechanisms through which MedDiet can modulate the presence of chronic diseases have been described [61]: (1) reducing chronic inflammation (a common mechanism in cardiovascular disease, metabolic disease, diabetes or cancer); (2) improving the HDL reverse cholesterol transport, increase its efflux and change the ability of HDL to esterify cholesterol; (3) induce autophagy response by means of polyphenols (present in grapes, wine, nuts and virgin olive oil); (4) having an effect over our genes and natural microbiota, studied by the omics: transcriptomics (MedDiet can change gene expression related to specific diseases), epigenomics (genome modifications without DNA changes but with changes in gene expression), genomics (genomic influence on the MedDiet effects), metabolomics (MedDiet has good effects on the microbiota or the metabolic network) and proteomics. Other potential mechanisms have been addressed by Martínez-González et al in the context of omics studies on the MedDiet conducted within the PREDIMED trial [61].

## 4. Conclusions

In our review of the research conducted during the last 18 years in the SUN prospective cohort, we can conclude that this study has provided sufficient longitudinal evidence that better adherence to the MedDiet was inversely associated with the risk of overall all-cause mortality. The inverse association was stronger for men. Considering both a high adherence to the MDS and a physically active lifestyle, a very strong reduction in the risk of all-cause mortality was found. A high intake of fruit and nuts and a low consumption of meat products were independently associated with this reduced risk and were driving a substantial part of the observed inverse association.

A better adherence to the MedDiet has also been associated with reduced fatal and non-fatal CVD and with a mitigation in the harmful effect of overweight/obesity on CVD. Again, fruits and nuts showed an independent inverse association with the incidence of CVD. Although a significant inverse association with the risk of developing hypertension was not found, a Mediterranean diet could contribute to the prevention of age-related changes in blood pressure (as a continuous variable) because the MDS was inversely associated with age-related changes in mean systolic and diastolic blood pressure after 6 years of follow-up, and in the PREDIMED intervention trial a reduction in diastolic (but not systolic) blood pressure was obtained with the MedDiet [62]. A direct association between alcohol intake and hypertension was found [41]. Additionally, a higher adherence to the MDS was associated with a significantly lower average heart rate.

A high adherence to the MDS was also associated with a strong reduction in the risk of type 2 diabetes (as confirmed later by several consistent prospective studies and trials [45,46]) and it can attenuate the adverse effects of obesity on the risk of type 2 diabetes. 

Better scores in the MDS were also associated with smaller weight gains (confirmed also in the PREDIMED long-term randomized trial [63]) although no association with the incidence of overweight or obesity was found in the SUN cohort. The highest level of adherence to the MDS was associated with a lower incidence of metabolic syndrome, as it was also confirmed in the PREDIMED trial and in large meta-analyses [64,65,66]. The large on-going PREDIMED-Plus trial using an energy-restricted Mediterranean diet and physical activity will provide first-level evidence on the role of the Mediterranean diet to prevent cardiovascular disease among overweight subjects with the metabolic syndrome ([67]; https://cordis.europa.eu/project/rcn/188509_en.html).

The SUN cohort has been a pioneer study in showing an inverse association between better conformity with a high-quality dietary pattern and the risk of depression [36], with a specific significant inverse dose-response relationship for fruit and nuts, legumes and the MUFA/SFA ratio. This finding was replicated later by other large cohorts [68,69,70]. A higher adherence to the MDS was also associated with higher lower scores in cognitive function and with a good mental health, as other cohorts and trials have also shown [71,72,73,74].

Finally, MedDiet may enhance fertility and reduce the risk of nephrolithiasis.

## 5. Limitations and Strengths

### 5.1. Limitations

Regarding the study population there are some limitations that should be acknowledged. Firstly, the SUN cohort is not representative of the general population, which could limit the external validity of our results. However, the restriction of the selection of university graduates could also improve the validity of our study as the high education and homogeneity of the participants can reduce the confounding related to social factors. Moreover, most cohorts and other analytical studies are usually non-representative, but the generalization should be based on biological plausibility. Secondly, the response rate of the SUN study is 10%. However, those accepting to participate are highly motivated participants which assure a high retention rate in the cohort (91%) [34]. Thirdly, our participants live in a Mediterranean country and consequently, for the majority the consumption of MedDiet components is high, even among those with a lower adherence. Finally, as it is a cohort in which most participants are young adults, there is a low number of new cases of chronic diseases/deaths which can limit the statistical power. 

With regards to the data, the SUN study is based on self-reported data. However, because of the high motivation and high educational level of the participants we can assume high quality of the information collected. In any case, some degree of misclassification could exist, but we would expect it to be nondifferential and therefore drive the association toward the null. Additionally, many of the variables measured have been validated. Another limitation that can be observed in this review is the variability across the MedDiet scores used, which can affect the results, particularly the dose-response relationships. However, a strength of the SUN cohort is the consistent use of the MDS proposed by Trichopoulou and its setting in a Mediterranean area, where olive oil is the most frequently consumed culinary fat. In any case, the problem of the variability in scores can also apply for most food patterns. Another possible drawback is that information on some Mediterranean eating aspects or foods, which could affect the findings, were not collected in the FFQ (e.g., aromatic herbs). Finally, most of the studies reviewed did not consider repeated measurements of the diet which could have led to some degree of misclassification bias (expected to be non-differential as commented above). 

### 5.2. Strengths

With regards to the study population, as previously explained the fact that all participants are university graduates adds quality to information collected and furthermore, as it is an educationally homogenous cohort the confounding for education and other social factors can be reduced. Anotherstrength concerns the fact that, as most participants are young adults, the cohort allows to evaluate the earliest steps in the pathophysiological mechanisms of the diseases analyzed. 

Regarding the study design, the SUN study has a large sample size, a prospective design (which allows to evaluate the temporal sequence of the associations), a long follow-up (soon some participants will answer to the Q18), has a good retention rate, it is coordinated in a single center (which simplifies the performance and avoids the errors) and many of the measurements have been validated.

Finally, all associations have been adjusted for a wide number of confounders and most of the studies have carried out sensitivity analyses.

Therefore, the SUN study, after 18 years of intense and productive work, has substantially contributed to the widespread acceptance in the academic world of the MedDiet as the ideal food pattern for the prevention of the major chronic diseases of the XXI century.

## Figures and Tables

**Table 1:** Items included in the baseline questionnaire (Q0) and in the follow-up questionnaires after 2, 4…, and 18 years (Q2 to Q18).

Number of Items Concerning Each Group of Characteristics in the Baseline and Follow-up Questionnaires											
	Q0	Q2	Q4	Q6	Q8	Q10	Q12	Q14	Q16	Q18	Qbrief
**SUN participation category**	-	1	-	-	-	-	-	-	-	-	-
**Sociodemographics**	10	1	1	1	2	2	1	3	3	2	1
Sex, birth date, residence, marital status, *n* children, *n* cohabitants, education, health-career, working status, religiosity											
**Anthropometrics**	4	1	1	4	4	2	1	1	5	2	1
Weight, weight change, birth weight, height, body image, waist circumference, hip circumference											
**Quality of life**	-	-	27	2	28	-	-	-	-	-	-
Health self-perception, health quality, pain											
**Diet**	136	9	9	15	-	136*	3	31	12	3	-
Dairy, meat, fish, eggs, vegetables, fruit, legumes, cereals, fats, sweets and pastries, nuts, miscellaneous, fiber											
Special diet, enriched foods											
Soft drinks, diet soft drinks, juices, energetic drinks											
Alcohol											
**Eating behaviors**	9	-	-	3	2	-	1	3	1	-	-
Eating out, fast-food, ready-made, snacking, cooking oils											
**Lifestyle**	44	3	4	2	4	-	-	18	7	3	-
Smoking, drinking, driving, physical activity, sedentarism, screen time, social networks, sleeping quality											
**Clinical data and Family/childhood history**	25	-	1	7	7	1	1	2	4	5	1
Heart rate, BP, cholesterol (total, LDL, HDL), TGs, glucose,											
Moles, sunburn, snoring, sleeping problems, memory loss, menstruation, pregnancy, breastfeeding, family records											
**Disease diagnosis**	32	34	33	34	35	35	42	44	47	51	37
-Diabetes, gestational diabetes, high cholesterol, high TGs, obesity											
-Hypertension, myocardial infarction, angina, by-pass, coronary angioplasty, CVA, paroxysmal tachycardia, atrial fibrillation, aortic aneurysm, CCF, thrombosis, arterial insufficiency, lung embolism,											
-Asthma, chronic bronchitis / emphysema											
-Traffic injuries, sport injury, hip fracture or other, (rheumatoid) arthritis, arthrosis, rheumatism, osteoporosis											
-Colon or rectal polyps, gastric/duodenal ulcer, gallstones											
-Kidney stones or renal colic											
-Cataracts, cataract surgery, myopia, hyperopia, presbyopia, astigmatism, glaucoma, retinal degenerative disease											
-Periodontal diseases											
-Sleep apnea, insomnia, anorexia or bulimia, anxiety, depression, memory loss, dementia, Alzheimer, Parkinson, migraine											
-Infertility, fibrocystic breast disease											
-Cancer or tumors											
-Surgery (hip, knee, gastric by-pass, coronary by-pass...)											
**Preventive screenings**	14	6	6	6	-	-	-	-	-		-
Healthcare center visit, GP examination, cholesterol, blood pressure, ECG, cardiovascular, respiratory, fecal occult blood, colonoscopy/sigmoidoscopy, dental, ophthalmologic, gynecologic, prostate											
**Preventive strategies**	3	3	3	3	3	-	-	-	-	-	-
Sunscreen, seat belt, airbag, helmet											
**Medications and supplements**	18	1	1	1	-	-	-	1	1	1	1
Analgesics, cholesterol lowering, antidiabetics, diuretics, cardiovascular, weight control, antidepressants, anxiolytics, insomnia, hormonal therapy, vitamins and minerals											
**Personality traits, feelings, and emotions**	3	-	9	-	9	-	-	-	25	29	-
Hard-working, competitive, stressed, aggressive, autonomous											
Feelings and emotions											

TG: triglycerides; CVA: cerebrovascular accident; CCF: congestive cardiac failure; GP: general practitioner; ECG: electrocardiogram. * Optional (online version of the questionnaire).

**Table 2:** Baseline characteristics of participants according to quintiles of adherence to the Mediterranean diet (MedDiet) in the SUN Project ^1^.

Adherence to the Mediterranean Diet Score (MDS-Trichopoulou: 0 to 9 Points)					
	0–2	3	4	5	6–9
*N*	3682	3571	4083	3669	4872
Profession, %					
Health professional	49.2	49.7	53.3	56.39	60.7
Marital status, %					
Married	50.9	50.2	50.8	50.0	49.1
Baseline BMI (kg/m^2^)	23.4 (3.6)	23.5 (3.6)	23.5 (3.5)	23.6 (3.6)	23.5 (3.5)
Physical activity during leisure time (MET-h/week)	22.2 (20.9)	25.3 (21.7)	26.9 (23.4)	28.1 (25.1)	32.3 (27.7)
Smoking status, %					
Never smoker	51.7	50.7	48.9	50.2	48.0
Current smoker	25.5	25.7	26.4	24.0	24.9
Former smoker	22.8	23.6	24.7	25.8	27.1
Total energy intake (kcal/day)	2,257.4 (800.7)	2,347.2 (842.0)	2,524.1 (917.6)	2,623.1 (974.8)	2,799.1 (1,068.4)
Carbohydrate intake, % E	40.4 (7.5)	42.0 (7.5)	43.1 (7.5)	44.3 (7.4)	46.1 (7.2)
Protein intake, % E	18.5 (3.9)	18.1 (3.8)	18.1 (3.5)	18.1 (3.3)	17.7 (3.1)
Fat intake, % E	39.7 (6.1)	37.9 (6.5)	36.8 (6.4)	35.6 (6.3)	33.9 (6.4)
SFAs, % E	15.2 (3.4)	13.6 (3.0)	12.6 (2.8)	11.6 (2.7)	10.2 (2.5)
MUFAs, % E	16.3 (3.4)	16.1 (4.1)	15.8 (3.8)	15.4 (3.7)	15.1 (3.7)
PUFAs, % E	5.3 (1.7)	5.2 (1.7)	5.2 (1.6)	5.1 (1.6)	5.1 (1.51)
Dietary fiber intake (g/day)	19.3 (8.3)	24.1 (10.8)	28.7 (13.8)	33.5 (15.3)	40.5 (18.3)
Alcohol intake (g/day)	4.5 (10.9)	6.1 (11.3)	6.8 (10.9)	7.3 (11.5)	8.7 (10.4)
Olive oil (g/day)	9.3 (9.6)	12.8 (12.5)	15.8 (14.2)	17.8 (14.8)	22.5 (16.1)
Vegetables (g/day)	332.9 (201.1)	433.5 (316.6)	532.6 (357.7)	627.8 (415.2)	767.7 (469.9)
Fruit and nuts (g/day)	194.7 (164.0)	274.9 (266.3)	352.4 (343.9)	429.7 (358.2)	543.7 (424.1)
Legumes (g/day)	16.7 (13.7)	20.9 (21.4)	23.7 (20.9)	26.2 (26.1)	29.6 (25.6)
Cereals (g/day)	76.7 (65.3)	91.1 (74.0)	106.8 (83.8)	119.3 (86.5)	144.4 (94.9)
Fish and seafood (g/day)	68.1 (63.8)	82.9 (52.1)	99.1 (70.8)	116.6 (88.9)	135.1 (90.8)
Dairy products (g/day)	467.3 (287.9)	446.1 (297.8)	450.1 (292.1)	438.5 (302.5)	422.7 (282.9)
Meat and meat products (g/day)	197.1 (95.3)	184.9 (92.2)	190.1 (106.5)	184.9 (108.2)	170.5 (108.3)
Soft-drinks (g/day)	71.5 (149.1)	71.3 (144.1)	70.1 (138.5)	68.2 (138.5)	64.6 (133.3)
Fast-food (g/day)	26.0 (35.2)	23.5 (22.7)	22.4 (23.2)	21.3 (23.5)	21.0 (30.4)

E: Energy; SFA: Saturated Fatty Acids; MUFA: Monounsaturated Fatty Acids; PUFA: Polyunsaturated Fatty Acids. ^1^: All values are means (SDs) unless otherwise indicated. Analyses were adjusted for age and sex using inverse probability weighting.

**Table 3:** Incident outcomes according to quintiles of adherence to the Mediterranean diet (MedDiet) in the SUN Project adjusted for age and sex using inverse probability weighting.

Adherence to the Mediterranean Diet Score (MDS-Trichopoulou: 0 to 9 Points) *					
	0–2	3	4	5	6–9
*N*	3682	3571	4083	3669	4872
Deaths, %					
CVD-deaths	0.1	0.5	0.3	0.3	0.2
Cancer-deaths	0.8	0.6	0.8	0.6	0.6
CVD events, %					
Non-fatal myocardial infarctions	0.6	0.4	0.3	0.3	0.2
Non-fatal strokes	0.2	0.4	0.2	0.3	0.1
Hypertension	9.9	8.9	8.7	8.0	8.1
Diabetes, %					
T2D	0.8	0.8	0.8	0.8	0.8
Overweight/Obesity, %	14.2	14.6	14.2	13.0	12.1
Metabolic syndrome, %	2.1	2.3	2.1	2.0	1.8
Depression, %	6.2	5.7	4.6	5.0	4.8
Difficulty conceiving, %	6.9	6.8	7.3	6.6	6.7
Nephrolithiasis, %	5.7	5.2	4.8	5.0	4.7

CVD: Cardiovascular Disease; T2D: Type 2 Diabetes. * Some comparisons of these rates will not exactly match the estimates reported in previous publications because: (a) there are no exclusions in this table; (b) the rates shown here are not adjusted for further residual confounding beyond sex and age; (c) new cases confirmed after our previous publications have been included in this updated table.

**Table 4:** Major findings on Mediterranean diet and health outcomes.

Publication	Study Population (*N*) and Recruitment Period	Follow-up	Exposure	Outcome (*n* of Events)	Results
**Mortality**					
Alvarez-Alvarez et al, 2017 [1]	*N* = 19,467 (Dec99–Feb13)	Median:10.3 years	MDS --------------------------------------- MDS+ physical activity	All-cause mortality. (*n* = 305) Deaths: 46.3% cancer, 20.6% CVD	MDS (6–9 vs. 0–2): HR = 0.79 (0.51–1.22), *p* trend = 0.018. +2 points: HR = 0.86 (0.73–1.00) ↑MDS + ↑PA: HR = 0.29 (0.13–0.65).
Zazpe et al., 2014 [30]	*N* = 16,008 (Dec99–Sept09)	Median: 6.9 years (0.10–12.9)	Empirically derived MedDiet * (rich in vegetables, fish and seafood, fruits, and olive oil)	All-cause mortality (*n* = 148) Deaths: 36.9%cancer, 18.1% CVD	MedDiet (T3 vs.T1): HR = 0.53 (0.34–0.84), *p* trend 0.01.
Domínguez et al., 2013 [37]	*N* = 9,109 (Dec99–Sept09)	Mean: 6.2 years (1.6–10.0)	MDS	Composite outcome (deaths = 131; diabetes = 58; CVD = 95)	MDS (+2 points): OR = 0.81 (0.69–0.95), *p* trend < 0.01
Martínez-González et al., 2012 [38]	*N* = 15,535 (Dec99–March09)	Mean: 6.8 years	MDS	Total mortality (*n* = 125) (20.8% CVD)	MDS (3–5 vs. 0–2): HR = 0.58 (0.34–0.99) MDS (6–9 vs. 0–2): HR = 0.38 (0.21–0.70) MDS (+ 2-point): HR = 0.72 (0.58–0.91), *p* trend = 0.006
**Cardiovascular disease**					
Eguaras S, et al., 2015 [39]	*N* = 19,065 (Dec99–Dec14)	Mean: 10.9 years	MDS	CVD (*n* = 152) (56 non-fatal myocardial infarctions, 30 non-fatal strokes and 66 CV deaths) Mitigation of consequences of obesity on CVD	MDS (7–9 vs. 0–2): HR = 0.47 (0.25–0.89), *p* trend = 0.029. MDS (+2 point): HR = 0.93 (0.89–0.98) MDS (<6/9): -BMI (25–30 vs. <25): HR = 1.44 (0.93–2.25) -BMI (>30 vs. <25): HR = 2.00 (1.04–3.83) MDS (≥6/9): -BMI (25–30 vs. <25): HR = 0.77 (0.35–1.67) -BMI (>30 vs. <25): HR = 1.15 (0.39–3.43)
Martínez-González et al., 2011 [40]	*N* = 13,609 (Dec99-Jan06)	Median: 4.9 years	MDS	CVD (*n* = 100) (68 coronary acute, 7 revascularization, and 32 strokes; 4 infarctions and 4 strokes).	MDS (7–9 vs. 0–2): HR CVD=0.41 (0.18–0.95), *p* trend = 0.07 XMDS (7–9 vs. 0–2): HR coronary = 0.42 (0.16–1.11), *p* trend = 0.04 MDS (+2-point): HR CVD = 0.80 (0.62–1.02) MDS (+2 point): HR coronary disease = 0.74 (0.55–0.99)
Núñez-Córdoba et al., 2009 [41]	*N* = 9,408 (Dec99–May05)	Median: 4.2 years (1.9–7.9)	MDS	Hypertension (*n* = 501) (≥140/≥90 or antihypertensive medication)	MDS (7–9 vs. 0–2): HR = 1.12 (0.79–1.60), *p* trend = 0.41 - Systolic blood pressure change: -3.1 mm Hg (−5.4, −0.8) - Diastolic blood pressure change: −1.9 mm Hg (−3.6, −0.1)
García-López et al., 2014 [42]	*N* = 15,863 (Dec99–Dec11)		MDS	Heart rate (HR) (bpm) (self-reported)	MDS (7–9 vs. 0–2): −2.2bpm (−3.1, −1.4), *p* trend < 0.001 +2 points:−0.77 bpm (−1.02,−0.51), *p* trend < 0.001
**Diabetes**					
Martínez-González et al., 2008 [44]	*N* = 13,380 (Dec99–Nov07)	Median: 4.4 years	MDS	Diabetes (additional-specific diabetes confirmation questionnaire) (*n* = 33)	MDS (3–6 vs. 0–2): RR = 0.41 (0.19 to 0.87) MDS (7–9 vs.0–2): RR = 0.17 (0.04 to 0.75) MDS (+2point): 0.65 (0.44 to 0.95), *p* trend = 0.04
Eguaras et al., 2017 [48]	*N* = 18,225 (Dec99–Dec15)	Median: 9.5 years	MDS	T2D associated with obesity (additional-specific diabetes confirmation questionnaire) (*n* = 136)	MDS (≤4): -BMI (25–30 vs. <25): HR = 4.07 (1.58–10.50) -BMI (>30 vs. <25): HR = 17.70 (6.29–49.78) MDS (>4): -BMI (25–30 vs. <25): HR = 3.13 (1.63–6.01) -BMI (>30 vs. <25): HR = 10.70 (4.98–22.99)
Donazar-Ezcurra et al., 2017 [31]	*N* = 3,455 (pregnant women) (Dec99-Mar13)	Mean:10.3 ± 3.3 years	Empirically derived MedDiet (high consumption of vegetables, fruits, fish, and non-processed foods)	Gestational diabetes (*n* = 173)	MedDiet (Q4 vs. Q1): OR = 1.08 (0.68–1.70)
**Weight change**					
Beunza et al., 2010 [49]	*N* = 10,376 (Dec99–Apr07)	Mean: 5.7 ± 2.2 years	MDS	-average yearly weight change (kg) -relevant weight gain (>3 or >5 kg/year) -overweight or obesity (≥25 kg/m^2^) -% of change from baseline weight (weight was assessed biennially)	MDS (6–9 vs. 0–3): -Average weight change: −0.059 (−0.111, −0.008) -Weight gain (≥3 or ≥5 kg) 2 year ≥3 kg: 0.80 (0.70, 0.92)/≥5 kg: 0.76 (0.62, 0.92) 4 year ≥3 kg: 0.80 (0.71, 0.91)/≥5 kg: 0.76 (0.64, 0.90) -Overweight/obesity: HR = 0.90 (0.75, 1.06) -% of change from baseline weight Lowest adherence: 1.21% (0.32, 2.11%) Highest adherence 1.10% (0.20, 1.99%) (linear)
Sánchez-Villegas et al., 2006 [50]	*N* = 6,319	28 months	MDS * * Calculated according to the tertiles distribution for the consumption of several components of the MedDiet instead of dichotomous considering the median.	-weight change-BMI change -overweight or obesity (*n* = 353)	Weight change (crude analysis) Q1: +0.73 kg; Q4:+ 0.45 kg, p trend = 0.016 BMI change (crude analysis) Q1: +0.26; Q4:+ 0.17, *p* trend = 0.026 Overweight/obesity: MDS (Q4 vs. Q1):OR = 0.90 (0.59–1.38), *p* trend = 0.739
**Metabolic syndrome**					
Tortosa et al., 2007 [54]	*N* = 2,563	Median: 74 months	MDS	Metabolic syndrome	Incidence (%): 2.6, 2.5 and 0.8 (0–2, 3–5, 6–9) MDS (3–5 vs. 0–2): OR = 0.80 (0.42–1.54) MDS (6–9 vs. 0–2): OR = 0.20 (0.06–0.63), *p* trend = 0.013
**Depression**					
Sánchez-Villegas et al., 2015 [55]	*N* = 15,093 (Dec99–June14)	Median: 8.5 years	MDS	Depression (physician diagnosis and/or antidepressant) (*n* = 1,051)	MDS (6–9 vs. 0–2):HR = 0.70 (0.58–0.85), *p* trend = 0.002 Possible *L*-shaped associations
Sánchez-Villegas et al., 2009 [56]	*N* = 10,094 (Dec99–May05)	Median: 4.4 years	MDS	Depression (physician diagnosis and/or antidepressant) (*n* = 480)	MDS (6–9 vs. 0–2): HR = 0.58 (0.44–0.77), *p* trend < 0.001. Fruit, nuts (Q5 vs. Q1): HR = 0.61 (0.45–0.82), *p* trend = 0.007 Legumes (Q5 vs. Q1): HR = 0.76 (0.57–1.00), *p* trend = 0.03 MUFA/SFA (Q5 vs. Q1): HR = 0.76 (0.56–1.02), *p* trend = 0.04
**Cognitive decline**					
Galbete et al., 2015 [29]	*N* = 823 (> 55 year) (Sub-study 2008–2010)	Mean: 6–8 years	MDS	Cognitive decline (difference final-Initial, points in TICS-m)	MDS (<4 vs. 7–9): Difference = −0.43 (−0.92 to 0.05). MDS (4–6 vs. 7–9): Difference = −0.62 (−1.07 to −0.18). MDS (0–6 vs. 7–9): Difference = −0.56 (−0.99 to −0.13).
**Quality of life**					
Ruano et al., 2013 [32]	*N* = 11,128	4 years	Empirically derived MedDiet (high in fruits, vegetables, and olive oil.)	Quality of life Self-perceived mental and physical (validated)	MedDiet (Q5 vs. Q1): +1.3 (physical) to +3.4 (for vitality). MedDiet (Q5 vs. Q1): *p* trend = 0.007
Henríquez-Sánchez et al., 2012 [59]	*N* = 11,015 (Dec99–May10)	4 years	MDS	Quality of life Self-perceived mental and physical (validated)	MDS (+1 point). Vitality: *β* = 0.50, 0.32–0.68 MDS (+1 point). General health: *β* = 0.45, 0.26–0.62
**Reproductive health**					
Toledo et al., 2011 [33]	*N* = 10,977 women (Dec99–Jul07)	---	Empirically derived MedDiet (high consumption of vegetables, fish, fruits, poultry, low-fat dairy products, and olive oil)	Difficulty conceiving	MedDiet (Q4 vs. Q1): OR = 0.56 (0.35–0.90), *p* trend = 0.01
**Nephrolithiasis**					
Leone et al., 2017 [60].	*N* = 16,094 (Dec99–Mar13)	Mean: 9.6 years	MDS	Nephrolithiasis (*n* = 735)	MDS (4–6 vs. 0–3): HR = 0.93 (0.79–1.09) MDS (7–9 vs. 0–3): HR = 0.64 (0.48–0.87); *p* trend = 0.01. Dairy (Q5 vs. Q1): HR = 0.78 (0.61–0.99), *p* trend = 0.01 Vegetables (Q5 vs. Q1): HR = 0.71 (0.55–0.90), *p* trend = 0.01 MUFA/SFA (Q5 vs. Q1): HR = 1.38 (1.08–1.76), *p* trend = 0.01

BMI: Body Mass Index; HR: Hazard Ratio; MDS: Mediterranean Diet Score (Trichopoulou); OR: Odds Ratio
